# 5-[(2-Methyl-4-nitro-1*H*-imidazol-1-yl)meth­yl]-1,3,4-thia­diazol-2-amine

**DOI:** 10.1107/S1600536813030821

**Published:** 2013-11-27

**Authors:** M. K. Usha, S. Madan Kumar, H. S. Vidyashree Jois, B. Kalluraya, N. K. Lokanath, D. Revannasiddaiah

**Affiliations:** a Department of Studies in Physics, University of Mysore, Manasagangotri, Mysore 570 006, India; b Department of Studies in Chemistry, Mangalore University, Mangalagangotri, Mangalore 574 199, India

## Abstract

In the title compound, C_7_H_8_N_6_O_2_S, the dihedral angle between the imidazole and thia­diazole rings is 70.86 (15)°. In the crystal, mol­ecules are linked into [10-1] chains by N—H⋯N hydrogen bonds, which incorporate centrosymmetric *R*_2_^2^(8) and *R*_2_^2^(18) loops. The chains are linked by C—H⋯O and C—H⋯N inter­actions, generating a three-dimensional network. Very weak π–π stacking [centroid–centroid distance = 3.901 (17) Å] is also observed.

## Related literature

For biological background, see: Dogan *et al.* (2002 ▶); Frank & Kalluraya (2005 ▶); Mullican *et al.* (1993 ▶). For related structures, see: Zama *et al.* (2013 ▶); Yin *et al.* (2012 ▶).
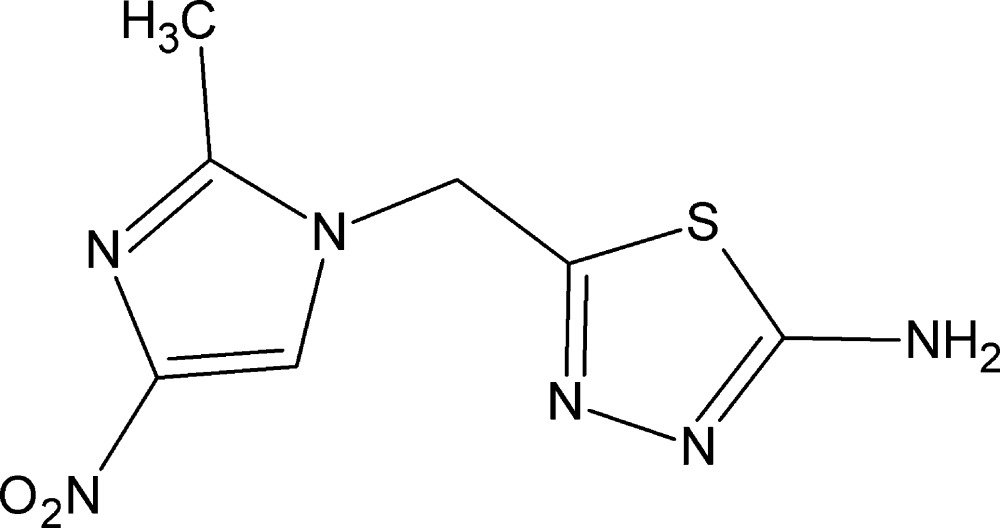


## Experimental

### 

#### Crystal data

C_7_H_8_N_6_O_2_S
*M* *_r_* = 240.26
Triclinic, 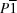

*a* = 7.8030 (15) Å
*b* = 8.2750 (16) Å
*c* = 8.3596 (16) Å
α = 100.945 (8)°
β = 92.379 (8)°
γ = 105.911 (7)°
*V* = 507.15 (17) Å^3^
*Z* = 2
Cu *K*α radiation
μ = 2.86 mm^−1^
*T* = 296 K
0.23 × 0.22 × 0.21 mm

#### Data collection

Bruker X8 Proteum diffractometer
Absorption correction: multi-scan (*SADABS*; Bruker, 2013 ▶) *T*_min_ = 0.559, *T*_max_ = 0.585
5433 measured reflections
1640 independent reflections
1560 reflections with *I* > 2σ(*I*)
*R*_int_ = 0.038

#### Refinement

*R*[*F* ^2^ > 2σ(*F* ^2^)] = 0.084
*wR*(*F* ^2^) = 0.206
*S* = 1.10
1640 reflections
147 parameters
H-atom parameters constrained
Δρ_max_ = 0.80 e Å^−3^
Δρ_min_ = −0.65 e Å^−3^

### 

Data collection: *APEX2* (Bruker, 2013 ▶); cell refinement: *SAINT* (Bruker, 2013 ▶); data reduction: *SAINT*; program(s) used to solve structure: *SHELXS97* (Sheldrick, 2008 ▶); program(s) used to refine structure: *SHELXL97* (Sheldrick, 2008 ▶); molecular graphics: *Mercury* (Macrae *et al.*, 2008 ▶); software used to prepare material for publication: *PLATON* (Spek, 2009 ▶).

## Supplementary Material

Crystal structure: contains datablock(s) global, I. DOI: 10.1107/S1600536813030821/hb7159sup1.cif

Structure factors: contains datablock(s) I. DOI: 10.1107/S1600536813030821/hb7159Isup2.hkl

Supplementary material file. DOI: 10.1107/S1600536813030821/hb7159Isup3.cml

Additional supplementary materials:  crystallographic information; 3D view; checkCIF report

## Figures and Tables

**Table 1:** Hydrogen-bond geometry (Å, °)

*D*—H⋯*A*	*D*—H	H⋯*A*	*D*⋯*A*	*D*—H⋯*A*
N3—H3*A*⋯N1^i^	0.86	2.15	2.996 (4)	169
N3—H3*B*⋯N5^ii^	0.86	2.26	3.033 (4)	150
C3—H3*D*⋯O1^iii^	0.97	2.46	3.100 (4)	123
C4—H4⋯N2^iv^	0.93	2.51	3.296 (4)	142
C7—H7*C*⋯O2^v^	0.96	2.57	3.445 (4)	152

Symmetry codes: (i) 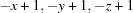; (ii) 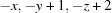; (iii) 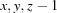; (iv) 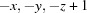; (v) 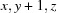.

**Fig. 1.**
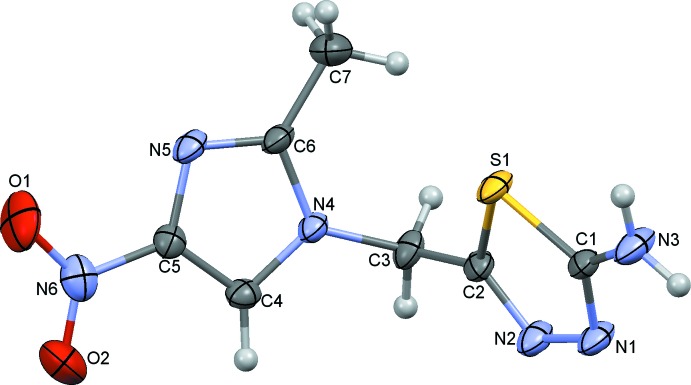
ORTEP diagram of the title compound with 50% probability ellipsoids.

**Fig. 2.**
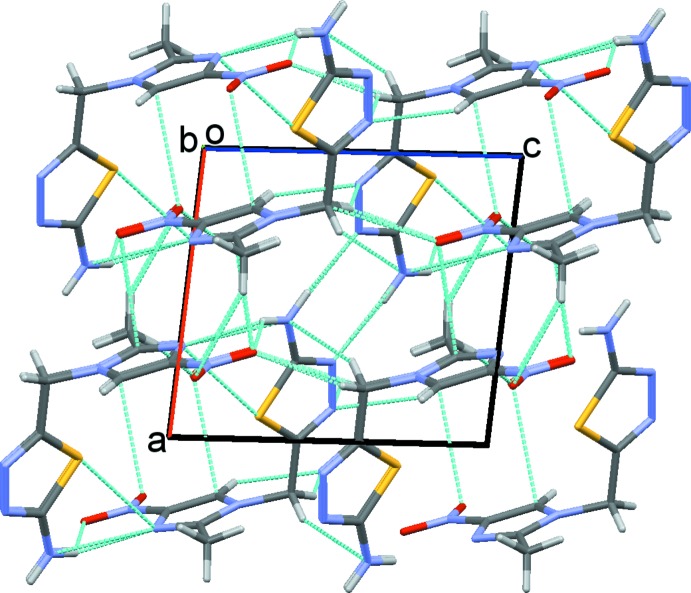
Packing diagram of molecule, viewed along b axis.

## supplementary crystallographic information

### 1. Comment

Five-membered aromatic systems having three hetero atoms at symmetrical positions have been studied because of their interesting physiological properties (Dogan *et al.*, 2002). It is also well established that various derivatives of 1,3,4-thiadiazoles exhibit broad spectrum of pharmacological properties such as antibacterial, antifungal (Frank & Kalluraya, 2005) and anti inflammatory (Mullican *et al.*, 1993) activities. As part of our studies in this area, we now report the synthesis and structure of the title compound.

The bond distances in the title compound are comparable to related structures methyl 2-(2-methyl-4-nitro-1*H*-imidazol-1-yl)acetate (Zama *et al.*, 2013) and 5-({[(*E*)-Benzylideneamino]oxy}methyl)-1,3,4-thiadiazol-2-amine (Yin *et al.*, 2012). The *ORTEP* of the title compound is shown (Fig. 1) and the dihedral angle between imidazol and thiadiazol ring is 70.86 (15)°. In the crystal, the molecules are connected by hydrogen bonds (Table 2) N3—H3A···N1, N3—H3B···N5, C4—H4···N2 with *R*_2_^2^(8), *R*_2_^2^(18) and *R*_2_^2^(12) ring motifs, respectively. The C3—H3D···O1 and C7—H7···O2 intermolecular hydrogen bonds generate continuous chains along *c-*axis and *b-*axis, respectively. Also, π–π interactions (*Cg1···Cg1*)[with minimum centroid-centroid distance 3.901 (17) Å] are observed. The centroid *Cg*1 is S1/C1/N1/N2/C2 and *Cg*2 is N4/C4/C5/N5/C6. The packing of the molecules show three-dimensional architecture (Fig. 2).

### 2. Experimental

A mixture of 2-methyl-4-nitro-1-imidazo thiosemicarbazide (1 mmol) and conc. sulfuric acid (1 ml) was heated under reflux for 2–3 h. The resulting solution was cooled, poured into crushed ice and treated with sodium carbonate to pH 6. The precipitate was collected by filtration and washed with water. The solid formed was filtered and recrystallized from ethanol-DMF mixture to yield red blocks (Melting point 251 °C).

### 3. Refinement

The H atoms were placed in calculated positions (C–H = 0.93-0.97Å and N–H = 0.86 Å), and refined as riding on their parent C and N atoms with U_iso_(H) = 1.2 U_eq_(C,N).

### Crystal data

**Table:**

C_7_H_8_N_6_O_2_S	*Z* = 2
*M**_r_* = 240.26	*F*(000) = 248
Triclinic, *P*1	*D*_x_ = 1.573 Mg m^−^^3^
Hall symbol: -P 1	Cu *K*α radiation, λ = 1.54178 Å
*a* = 7.8030 (15) Å	Cell parameters from 1640 reflections
*b* = 8.2750 (16) Å	θ = 5.4–65.5°
*c* = 8.3596 (16) Å	µ = 2.86 mm^−^^1^
α = 100.945 (8)°	*T* = 296 K
β = 92.379 (8)°	Block, red
γ = 105.911 (7)°	0.23 × 0.22 × 0.21 mm
*V* = 507.15 (17) Å^3^	

### Data collection

**Table:**

Bruker X8 Proteum diffractometer	1640 independent reflections
Radiation source: Bruker MicroStar microfocus rotating anode	1560 reflections with *I* > 2σ(*I*)
Helios multilayer optics monochromator	*R*_int_ = 0.038
Detector resolution: 10.7 pixels mm^-1^	θ_max_ = 64.5°, θ_min_ = 5.4°
φ and ω scans	*h* = −9→9
Absorption correction: multi-scan (*SADABS*; Bruker, 2013)	*k* = −9→9
*T*_min_ = 0.559, *T*_max_ = 0.585	*l* = −9→9
5433 measured reflections	

### Refinement

**Table:**

Refinement on *F*^2^	Secondary atom site location: difference Fourier map
Least-squares matrix: full	Hydrogen site location: inferred from neighbouring sites
*R*[*F*^2^ > 2σ(*F*^2^)] = 0.084	H-atom parameters constrained
*wR*(*F*^2^) = 0.206	*w* = 1/[σ^2^(*F*_o_^2^) + (0.170*P*)^2^ + 0.1569*P*] where *P* = (*F*_o_^2^ + 2*F*_c_^2^)/3
*S* = 1.10	(Δ/σ)_max_ < 0.001
1640 reflections	Δρ_max_ = 0.80 e Å^−^^3^
147 parameters	Δρ_min_ = −0.65 e Å^−^^3^
0 restraints	Extinction correction: *SHELXL97* (Sheldrick, 2008), FC^*^=KFC[1+0.001XFC^2^Λ^3^/SIN(2Θ)]^-1/4^
Primary atom site location: structure-invariant direct methods	Extinction coefficient: 0.128 (15)

### Special details

**Table:**

Geometry. Bond distances, angles *etc*. have been calculated using the rounded fractional coordinates. All su's are estimated from the variances of the (full) variance-covariance matrix. The cell e.s.d.'s are taken into account in the estimation of distances, angles and torsion angles
Refinement. Refinement on *F*^2^ for ALL reflections except those flagged by the user for potential systematic errors. Weighted *R*-factors *wR* and all goodnesses of fit *S* are based on *F*^2^, conventional *R*-factors *R* are based on *F*, with *F* set to zero for negative *F*^2^. The observed criterion of *F*^2^ > σ(*F*^2^) is used only for calculating -*R*-factor-obs *etc*. and is not relevant to the choice of reflections for refinement. *R*-factors based on *F*^2^ are statistically about twice as large as those based on *F*, and *R*-factors based on ALL data will be even larger.

### Fractional atomic coordinates and isotropic or equivalent isotropic displacement parameters (Å^2^)

**Table:**

	*x*	*y*	*z*	*U*_iso_*/*U*_eq_	
S1	0.07927 (9)	0.48240 (8)	0.72687 (8)	0.0272 (3)	
O1	−0.3024 (4)	−0.0160 (4)	1.2300 (3)	0.0530 (10)	
O2	−0.2117 (4)	−0.1962 (3)	1.0555 (4)	0.0520 (10)	
N1	0.2714 (3)	0.3731 (3)	0.5159 (3)	0.0301 (8)	
N2	0.1108 (3)	0.2446 (3)	0.4968 (3)	0.0292 (8)	
N3	0.4148 (4)	0.6476 (3)	0.6733 (3)	0.0346 (9)	
N4	−0.2299 (3)	0.1372 (3)	0.7565 (3)	0.0217 (7)	
N5	−0.3183 (3)	0.1775 (3)	1.0046 (3)	0.0253 (8)	
N6	−0.2558 (4)	−0.0653 (3)	1.0942 (3)	0.0327 (9)	
C1	0.2758 (4)	0.5055 (4)	0.6315 (3)	0.0229 (8)	
C2	−0.0004 (4)	0.2824 (3)	0.5963 (3)	0.0224 (8)	
C3	−0.1855 (4)	0.1641 (4)	0.5939 (3)	0.0257 (9)	
C4	−0.2020 (4)	0.0071 (3)	0.8234 (3)	0.0230 (8)	
C5	−0.2577 (4)	0.0367 (3)	0.9743 (3)	0.0228 (8)	
C6	−0.3017 (4)	0.2373 (3)	0.8694 (3)	0.0237 (8)	
C7	−0.3577 (5)	0.3853 (4)	0.8382 (4)	0.0396 (11)	
H3A	0.50960	0.65610	0.62280	0.0420*	
H3B	0.40920	0.73040	0.75070	0.0420*	
H3C	−0.27200	0.21210	0.54820	0.0310*	
H3D	−0.19450	0.05410	0.52310	0.0310*	
H4	−0.15570	−0.08100	0.77630	0.0280*	
H7A	−0.48610	0.35570	0.82610	0.0600*	
H7B	−0.31210	0.41570	0.73980	0.0600*	
H7C	−0.31140	0.48100	0.92860	0.0600*	

### Atomic displacement parameters (Å^2^)

**Table:**

	*U*^11^	*U*^22^	*U*^33^	*U*^12^	*U*^13^	*U*^23^
S1	0.0282 (6)	0.0236 (6)	0.0256 (6)	0.0051 (4)	0.0134 (3)	−0.0037 (4)
O1	0.079 (2)	0.0551 (17)	0.0176 (12)	0.0078 (14)	0.0032 (11)	0.0072 (11)
O2	0.0665 (19)	0.0387 (15)	0.0623 (17)	0.0249 (13)	0.0124 (14)	0.0226 (13)
N1	0.0320 (14)	0.0244 (13)	0.0298 (14)	0.0055 (10)	0.0161 (10)	−0.0031 (10)
N2	0.0321 (14)	0.0231 (13)	0.0280 (13)	0.0044 (10)	0.0135 (11)	−0.0022 (10)
N3	0.0309 (15)	0.0277 (14)	0.0377 (16)	0.0028 (11)	0.0177 (11)	−0.0062 (11)
N4	0.0245 (12)	0.0199 (12)	0.0196 (12)	0.0057 (9)	0.0085 (9)	0.0012 (9)
N5	0.0274 (13)	0.0215 (13)	0.0225 (13)	0.0038 (10)	0.0102 (9)	−0.0031 (10)
N6	0.0326 (15)	0.0302 (15)	0.0294 (15)	−0.0002 (11)	−0.0024 (11)	0.0068 (11)
C1	0.0268 (15)	0.0227 (14)	0.0208 (14)	0.0090 (11)	0.0100 (11)	0.0040 (11)
C2	0.0298 (16)	0.0209 (14)	0.0169 (13)	0.0076 (12)	0.0079 (11)	0.0030 (10)
C3	0.0281 (16)	0.0284 (16)	0.0169 (14)	0.0037 (12)	0.0066 (11)	0.0013 (11)
C4	0.0233 (14)	0.0169 (14)	0.0263 (15)	0.0046 (11)	0.0043 (11)	0.0001 (11)
C5	0.0238 (14)	0.0194 (14)	0.0213 (14)	0.0018 (11)	0.0026 (11)	0.0015 (11)
C6	0.0234 (14)	0.0208 (14)	0.0256 (15)	0.0059 (11)	0.0116 (11)	0.0001 (11)
C7	0.046 (2)	0.0322 (17)	0.049 (2)	0.0214 (15)	0.0195 (16)	0.0104 (15)

### Geometric parameters (Å, º)

**Table:**

S1—C1	1.739 (3)	N6—C5	1.430 (3)
S1—C2	1.735 (3)	N3—H3A	0.8600
O1—N6	1.236 (4)	N3—H3B	0.8600
O2—N6	1.215 (4)	C2—C3	1.503 (4)
N1—N2	1.384 (3)	C4—C5	1.352 (4)
N1—C1	1.308 (4)	C6—C7	1.471 (4)
N2—C2	1.286 (4)	C3—H3C	0.9700
N3—C1	1.340 (4)	C3—H3D	0.9700
N4—C3	1.459 (4)	C4—H4	0.9300
N4—C4	1.366 (4)	C7—H7A	0.9600
N4—C6	1.376 (4)	C7—H7B	0.9600
N5—C5	1.358 (4)	C7—H7C	0.9600
N5—C6	1.315 (3)		
			
C1—S1—C2	86.83 (14)	N4—C4—C5	103.6 (2)
N2—N1—C1	112.7 (2)	N6—C5—C4	125.5 (3)
N1—N2—C2	112.9 (2)	N5—C5—N6	121.4 (2)
C3—N4—C4	124.6 (2)	N5—C5—C4	113.1 (2)
C3—N4—C6	127.0 (2)	N4—C6—N5	110.2 (2)
C4—N4—C6	108.3 (2)	N4—C6—C7	124.1 (2)
C5—N5—C6	104.7 (2)	N5—C6—C7	125.7 (3)
O1—N6—O2	124.2 (3)	N4—C3—H3C	109.00
O1—N6—C5	117.7 (3)	N4—C3—H3D	109.00
O2—N6—C5	118.1 (3)	C2—C3—H3C	109.00
C1—N3—H3B	120.00	C2—C3—H3D	109.00
H3A—N3—H3B	120.00	H3C—C3—H3D	108.00
C1—N3—H3A	120.00	N4—C4—H4	128.00
N1—C1—N3	124.6 (3)	C5—C4—H4	128.00
S1—C1—N3	122.1 (2)	C6—C7—H7A	109.00
S1—C1—N1	113.3 (2)	C6—C7—H7B	109.00
S1—C2—N2	114.3 (2)	C6—C7—H7C	109.00
S1—C2—C3	123.6 (2)	H7A—C7—H7B	110.00
N2—C2—C3	122.1 (2)	H7A—C7—H7C	109.00
N4—C3—C2	112.6 (2)	H7B—C7—H7C	109.00

### Hydrogen-bond geometry (Å, º)

**Table:**

*D*—H···*A*	*D*—H	H···*A*	*D*···*A*	*D*—H···*A*
N3—H3*A*···N1^i^	0.86	2.15	2.996 (4)	169
N3—H3*B*···N5^ii^	0.86	2.26	3.033 (4)	150
C3—H3*D*···O1^iii^	0.97	2.46	3.100 (4)	123
C4—H4···N2^iv^	0.93	2.51	3.296 (4)	142
C7—H7*C*···O2^v^	0.96	2.57	3.445 (4)	152

Symmetry codes: (i) −*x*+1, −*y*+1, −*z*+1; (ii) −*x*, −*y*+1, −*z*+2; (iii) *x*, *y*, *z*−1; (iv) −*x*, −*y*, −*z*+1; (v) *x*, *y*+1, *z*.
